# Fe_2_O_3_–MgO–CuO@MWCNT Nanocomposite for Highly Sensitive Electrochemical Detection of l‑Cysteine at Low Potential in Biological Samples

**DOI:** 10.1021/acsomega.6c05509

**Published:** 2026-10-14

**Authors:** Danilo B. Ribeiro, Carolina A. Carvalho, Natilene M. Brito, Flavio S. Damos, Rita C. S. Luz

**Affiliations:** † 37892 Universidade Federal do Maranhão, Avenida dos Portugueses, No 1966, Bacanga, São Luís, Maranhão CEP 65080-805, Brasil; ‡ Instituto Federal de Educação, Ciência e Tecnologia do Maranhão, Avenida Getúlio Vargas, No 04, Monte Castelo, São Luís, Maranhão CEP 65030-005, Brasil

## Abstract

A Fe_2_O_3_–MgO–CuO composite integrated with multiwalled carbon nanotubes (MWCNTs) was employed to develop a platform for the indirect electrochemical detection of l-cysteine (l-CYS). The material was characterized by XRD, FTIR, SEM, EDS, and EIS, confirming the formation of a hybrid nanostructure. After optimization of the experimental parameters, the modified glassy carbon electrode exhibited a well-defined cathodic response at −0.25 V vs Ag/AgCl (saturated KCl). Although l-CYS undergoes electrochemical oxidation at positive potentials (approximately +0.25 V), quantitative analysis was based on the effect of the presence of l-CYS on the cathodic response of the modified electrode. The determination of the analyte was performed using linear sweep voltammetry, yielding two linear response ranges of 0.5–100 μmol L^–1^ (R^2^ = 0.992) and 100–1000 μmol L^–1^ (R^2^ = 0.996). The lowest detectable concentration of l-CYS with the Fe_2_O_3_–MgO–CuO@MWCNT/GCE electrode was 0.5 μmol L^–1^. The sensor exhibited high selectivity, excellent repeatability (RSD = 1.58%), and reproducibility (RSD = 1.82%). The sensor was evaluated for l-CYS determination in urine and breast milk samples, providing satisfactory recovery values.

## Graphical abstract

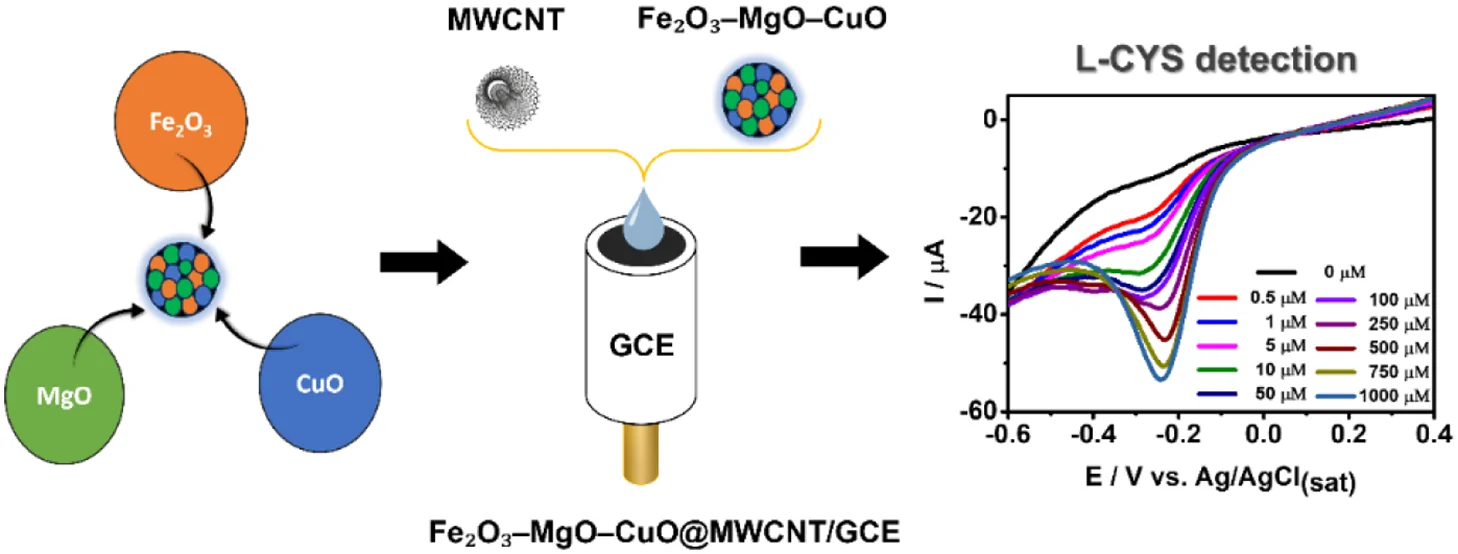

## Introduction

l-cysteine (l-CYS) is a biologically relevant amino acid that plays a crucial role in several metabolic processes, including protein structure, enzymatic activity, and cellular redox balance. Owing to the presence of a thiol group (−SH), l-CYS is directly involved in antioxidant defense mechanisms and acts as a precursor to glutathione, a key molecule in protecting cells against oxidative stress. Its thiol functionality also enables metal-ion binding, enzymatic catalysis, and participation in redox signaling pathways, making l-CYS an important biomarker in biological systems. Abnormal l-CYS levels have been associated with various pathological conditions, including cardiovascular and neurodegenerative diseases and cancer.^1–3^

Furthermore, l-cysteine is widely used in the pharmaceutical, food, and cosmetic industries, increasing the demand for reliable analytical methods for its quantification. Consequently, the accurate determination of l-CYS in biological matrices is important for clinical diagnosis, disease monitoring, and the investigation of pathological conditions. Several analytical techniques have been employed for l-CYS determination, including chromatographic, spectroscopic, and colorimetric methods.^4–9^ Although these approaches provide high sensitivity and reliability, they often require sophisticated instrumentation, relatively high costs, and complex sample preparation. In contrast, electrochemical methods offer advantages such as high sensitivity, low cost, operational simplicity, and potential for miniaturization, making them attractive for rapid and routine analyses.^10^

Despite these advantages, the direct electrochemical determination of l-CYS at conventional electrodes is challenging because of its relatively slow electron-transfer kinetics and the high overpotential generally required for its oxidation. These characteristics can limit sensitivity and increase the possibility of interference from other electroactive species. Therefore, the modification of electrode surfaces with electrocatalytically active materials has become an effective strategy to improve electron-transfer processes, increase the electrochemically accessible surface area, and enable l-CYS detection at lower potentials.^10–12^

Nanostructured materials have been widely investigated as electrode modifiers owing to their high surface area and favorable catalytic properties. Multimetallic systems are particularly interesting because the incorporation of different components can modify surface composition, electrochemically accessible sites, and charge-transfer characteristics at the electrode/electrolyte interface.^13–16^ Among these materials, trimetallic-based composites have attracted considerable attention due to their tunable physicochemical properties. In addition, trimetallic systems can offer synergistic effects and greater compositional versatility, which may lead to enhanced performance compared with their monometallic or bimetallic counterparts.^13,14^ CuO, Fe_2_O_3_, and MgO possess distinct electronic, surface, and physicochemical characteristics,^17–20^ and their integration may therefore modify the interfacial properties of the resulting material compared with the corresponding individual or binary systems.

Copper oxide-based materials have previously been investigated for cysteine determination. For example, a Cu_2_O-modified carbon paste electrode showed an electrocatalytic response toward cysteine, demonstrating the electrochemical activity of copper oxide-based surfaces toward this analyte.^21^ Fe_2_O_3_/CuO hybrid structures have also been investigated as electrode materials, showing that the combination of these oxides can modify the electrochemical behavior of the resulting material.^22^ In addition, CuO/MgO nanocomposites have been reported, demonstrating that the incorporation of MgO can influence the structural, electrical, and optical properties of CuO-based materials.^20^ Although these studies demonstrate the relevance of the individual and binary oxide systems, they do not establish whether the integration of Fe_2_O_3_, MgO, and CuO can produce a distinct electrochemical response toward l-CYS.

Fe_2_O_3_–MgO–CuO trimetallic oxide nanocomposites have previously been synthesized and characterized, with the study mainly focusing on their structural and optical properties.^19^ However, to the best of our knowledge, their application as an electrochemical sensing platform for l-CYS determination has not been previously investigated. Thus, the interest in the present material does not rely on claiming the first synthesis of Fe_2_O_3_–MgO–CuO, but rather on investigating its electrochemical properties and evaluating whether integration of the three oxide phases can modify the electrode/electrolyte interface and the resulting response toward l-CYS. This approach is particularly relevant because improved analytical performance alone does not demonstrate a specific contribution of a multicomponent architecture; such a contribution was evaluated through appropriate control electrodes and electrochemical characterization.

Carbon-based nanomaterials, particularly multiwalled carbon nanotubes (MWCNTs), are widely used in electrochemical sensing because of their excellent electrical conductivity, high surface area, and chemical stability.^23–25^ The incorporation of MWCNTs into metal oxide-based composites can provide conductive pathways that facilitate electron transport between electroactive components and the electrode surface. In this sense, carbon-based hybrid materials have been successfully investigated for l-CYS determination, including carbon-supported metal and metal oxide-based systems. In addition, MWCNTs can contribute to the dispersion of metal oxide particles, reducing agglomeration and increasing the accessibility of active sites. Moreover, their highly conductive network can favor a lower charge-transfer resistance and enhanced electron-transfer kinetics, resulting in improved analytical performance.^26–29^

Despite the progress in l-CYS electrochemical sensing, the Fe_2_O_3_–MgO–CuO trimetallic oxide has not, to the best of our knowledge, been investigated for this application. More importantly, it remains unclear whether a trimetallic system based on MgO, Fe_2_O_3_ and CuO produces electrochemical characteristics that differ from those of the individual oxides and the corresponding binary systems.

In this work, a Fe_2_O_3_–MgO–CuO trimetallic oxide integrated with MWCNTs was employed in an electrochemical sensing platform for l-CYS determination. The proposed material was investigated using appropriate control electrodes and electrochemical characterization to evaluate the contribution of the trimetallic architecture and, in particular, the effect of MgO incorporation into the Fe_2_O_3_–CuO system. In the present work, Nafion was used primarily to improve the stability and adhesion of the Fe_2_O_3_–MgO–CuO/MWCNT film on the glassy carbon electrode (GCE) surface due to its chemical stability, film-forming ability, and adhesion to electrode surfaces.^30,31^ Thus, the electrochemical behavior of the modified electrodes was examined to establish the relationship between material composition, interfacial properties, and l-CYS response. The proposed platform was further evaluated for l-CYS determination in biological samples, demonstrating its potential for electrochemical sensing applications.

## Materials and Methods

### Reagents and Solutions

All chemicals were of analytical grade and used without further purification. Magnesium nitrate hexahydrate (Mg­(NO_3_)_2_·6H_2_O, 97%), iron nitrate nonahydrate (Fe­(NO_3_)_3_·9H_2_O, 97%), copper nitrate trihydrate (Cu­(NO_3_)_2_·3H_2_O, 98%), absolute ethanol, sodium dihydrogen phosphate, sodium hydroxide, uric acid, citric acid, glucose, and urea were obtained from Isofar (Brazil). Nafion, l-cysteine, reduced glutathione and tryptophan were purchased from Sigma-Aldrich (USA). The pH of the phosphate buffer solution (0.1 mol L^−1^ PBS) was adjusted using a 10% (w/v) NaOH solution.

### Synthesis of Fe_2_O_3_–MgO–CuO Composite

The trimetallic oxide composite was synthesized using the sol–gel method, following a procedure adapted from the literature.^19^ Initially, three precursor solutions were prepared by dissolving Cu­(NO_3_)_2_·3H_2_O (1.691 g), Fe­(NO_3_)_3_·9H_2_O (2.825 g), and Mg­(NO_3_)_2_·6H_2_O (1.7945 g) separately in 10 mL of ethanol, maintaining a molar ratio of 1:1:1. Each solution was stirred for 10 min at room temperature to ensure complete dissolution and homogeneity. The solutions were then mixed and maintained under constant stirring at 80 °C for 70 min to promote the sol–gel transition. The resulting gel was dried to obtain a xerogel, which was ground into a fine powder and subsequently calcined at 800 °C for 90 min to ensure crystallization of the mixed oxides.

### Instrumentation

The crystalline structure of the synthesized materials was analyzed by X-ray diffraction (XRD) using a Bruker D8 Advance diffractometer equipped with Cu Kα radiation (40 kV, 40 mA), operating in the 2θ range from 20 to 70°. Fourier-transform infrared (FTIR) spectra were recorded using a Shimadzu IR Prestige-21 spectrophotometer. The samples were prepared as KBr pellets, and spectra were collected in the range of 400–4000 cm^–1^ with a resolution of 4 cm^–1^. The morphology of the material was examined by scanning electron microscopy (SEM). The micrographs were obtained using a Quanta 200 FEG-FEI microscope operated at an accelerating voltage of 10 kV. Electrochemical experiments were carried out in a conventional three-electrode system consisting of a working electrode (modified or bare GCE), an Ag/AgCl (sat. KCl) reference electrode, and a gold wire counter electrode. Measurements were performed using a μStat-i 400s potentiostat (Metrohm DropSens) controlled by DropView 8400 software.

### Preparation of the Modified Electrode

The glassy carbon electrode with a diameter of 3 mm and a geometric area of 0.071 cm^2^ was polished with a 0.3 μm alumina slurry and then rinsed with distilled water to remove surface impurities. A dispersion of MWCNTs was prepared by manually dispersing 2 mg of the material in 50 μL of distilled water. Separately, 2 mg of the Fe_2_O_3_–MgO–CuO composite was dispersed in 50 μL of distilled water. Equal volumes (20 μL) of the two dispersions were then manually mixed to obtain a uniform suspension. Next, 10 μL of the resulting suspension was drop-cast onto the GCE surface and dried at 50 °C (323 K) for 10 min to promote solvent evaporation and the formation of a stable modifier film. Afterward, 10 μL of a 0.25% Nafion solution (prepared in distilled water) was drop-cast onto the modified surface to improve film stability and adhesion. Finally, the Fe_2_O_3_–MgO–CuO@MWCNT/GCE electrode was dried at room temperature for 60 min before use.

### Human Urine and Breast Milk Samples Analysis

The applicability of the proposed sensor was evaluated in human urine and breast milk samples using the standard addition method. The samples were obtained from healthy volunteers after approval by the Research Ethics Committee of the University Hospital, Federal University of Maranhão (CEP Approval No. 2.982.185). Urine samples were diluted (1:20, v/v) in PBS prior to analysis. Breast milk samples were initially centrifuged at 3500 rpm for 15 min, and the collected supernatant was subsequently diluted (1:100, v/v) in PBS (pH 7.5).

Electrochemical measurements were carried out in a cell containing 10 mL of PBS. Initially, the response of the nonspiked sample was recorded. Subsequently, successive additions of standard l-CYS solution were performed to construct the calibration curves. Recovery studies were carried out at concentration levels of 50 and 100 μmol L^–1^.

## Results and Discussion

### Material Characterization

The crystalline structure of the synthesized trimetallic material was investigated by X-ray diffraction (XRD) to confirm the formation of Fe_2_O_3_–MgO–CuO phases. The diffraction pattern (Figure 1a) exhibited well-defined peaks at 2θ values of 30.12°, 32.56°, 35.47°, 36.94°, 38.80°, 43.08°, 48.72°, 53.48°, 56.99°, 58.30°, 61.56°, 62.57°, 66.18°, and 68.18°, indicating a polycrystalline structure. These reflections are consistent with previously reported Fe_2_O_3_–MgO–CuO composites, supporting the coexistence of monoclinic CuO, cubic MgO, and Fe_2_O_3_ phases, with no evidence of secondary crystalline phases within the XRD detection limits,^19^ suggesting the formation of a multiphase system with potential interfacial interactions.

**Figure 1:**
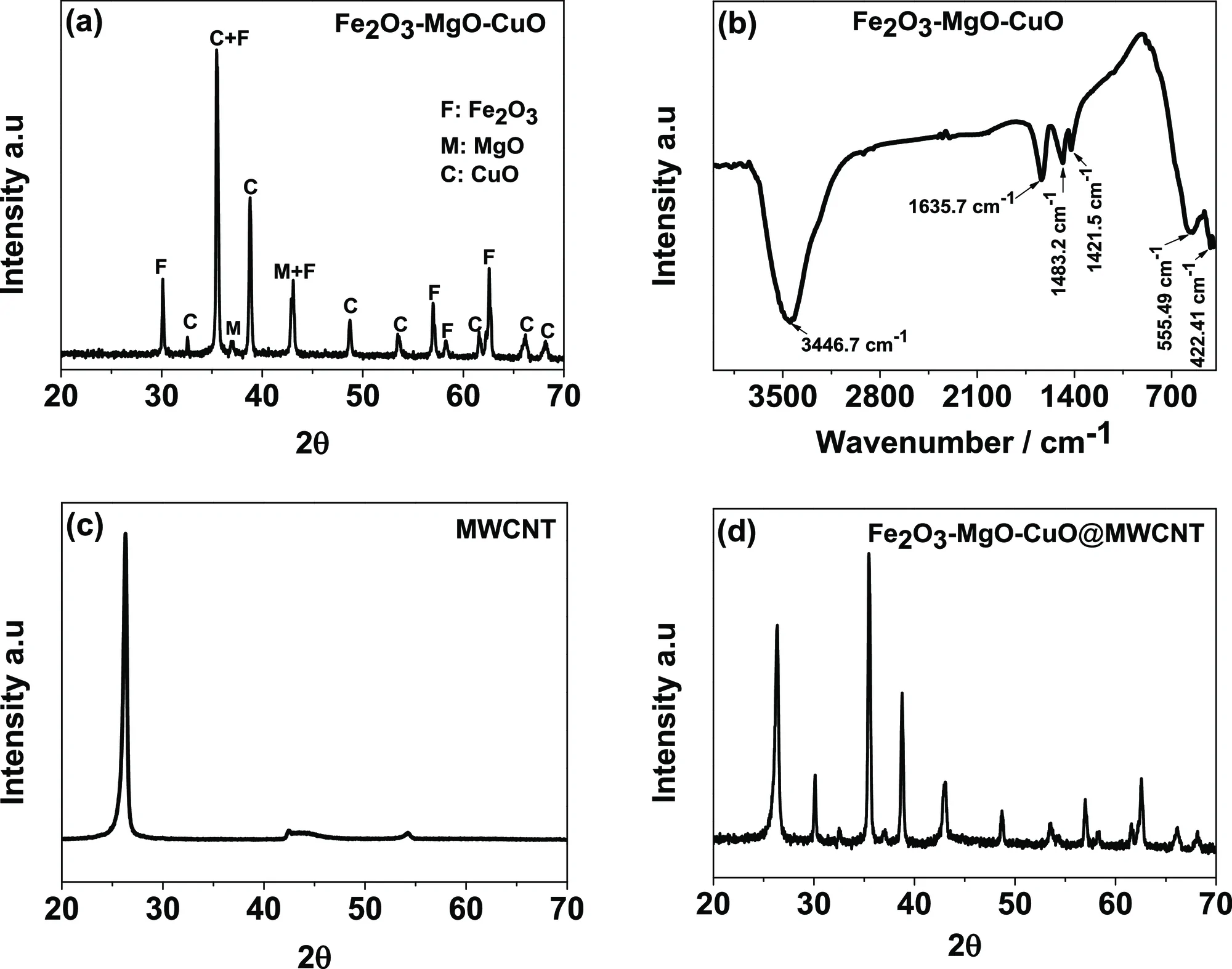
X-ray diffraction (XRD) patterns and Fourier-transform infrared (FTIR) spectrum of the Fe_2_O_3_–MgO–CuO composite (a, b), together with the XRD patterns of (c) MWCNTs and (d) the Fe_2_O_3_–MgO–CuO@MWCNT hybrid material.

The reflections can be indexed according to standard JCPDS cards of CuO (48-1548), Fe_2_O_3_ (39-1346), and MgO (45-0946), confirming the formation of the trimetallic oxide system. Specifically, peaks in the 35–39° and 48–53° regions are mainly attributed to the monoclinic CuO phase, while reflections around 43° and 62° correspond to cubic MgO. In addition, peaks located near 30°, 33–36°, and 57–63° are consistent with Fe_2_O_3_, further supporting its incorporation into the composite.

The FTIR spectrum (Figure 1b) also supports the successful formation of metal oxides-based composite. The broad and intense bands centered at 3446.7 cm^–1^ and 1635.6 cm^–1^ are assigned to the stretching and bending vibrations of O–H groups, respectively, indicating the presence of surface hydroxyl species and/or physisorbed water molecules.^32^

Weak bands observed at 1483.2 and 1421.5 cm^–1^ correspond to vibrational modes of carbonate-like species adsorbed on the oxides surface. This is a commonly observed phenomenon for metal oxides exposed to ambient CO_2_ and does not compromise the structural integrity of the composite. The most diagnostic features for the desired inorganic phase are observed at 555.49 cm^–1^ and 422.41 cm^–1^, which are attributed to metal–oxygen (M–O) stretching vibrations of Fe–O/Cu–O and Mg–O networks, respectively.^33–35^ Collectively, these spectroscopic results provide additional evidence for the formation of the intended trimetallic oxide composite comprising Fe, Mg, and Cu integrated into a single inorganic material.

The crystalline structure of MWCNTs was evaluated by X-ray diffraction (Figure 1c), showing a prominent peak at 2θ ≈ 26.2° ((002) plane) and a less intense reflection around 43° ((100)/(101) planes), confirming the preservation of their graphitic structure after processing.^36,37^ For the hybrid material (Figure 1d), the diffraction pattern showed the coexistence of MWCNT and metal oxide peaks, confirming the successful formation of the hybrid without disrupting the nanotube structure. Slight peak broadening and reduced intensity were observed, likely due to decreased crystallite size, dispersion of metal oxides on the MWCNT surface, and interfacial interactions, consistent with effective structural integration, which is expected to enhance electrical conductivity and promote faster electron transfer, contributing to improved electrochemical performance.

The morphology of the materials was investigated by field-emission scanning electron microscopy (FEG-SEM) (Figure 2). The MWCNTs (Figure 2a) exhibit typical entangled tubular structures, forming a highly interconnected three-dimensional conductive network with a high aspect ratio. In contrast, the Fe_2_O_3_–MgO–CuO composite displays aggregates composed of nearly spherical to irregular particles with rough surfaces (Figure 2b). These aggregates are relatively homogeneously distributed, forming a porous structure that may facilitate electrolyte diffusion and improve the accessibility of electroactive species. The absence of distinct segregated phases at the SEM scale suggests effective integration among the metal oxides, in agreement with the XRD results.

**Figure 2:**
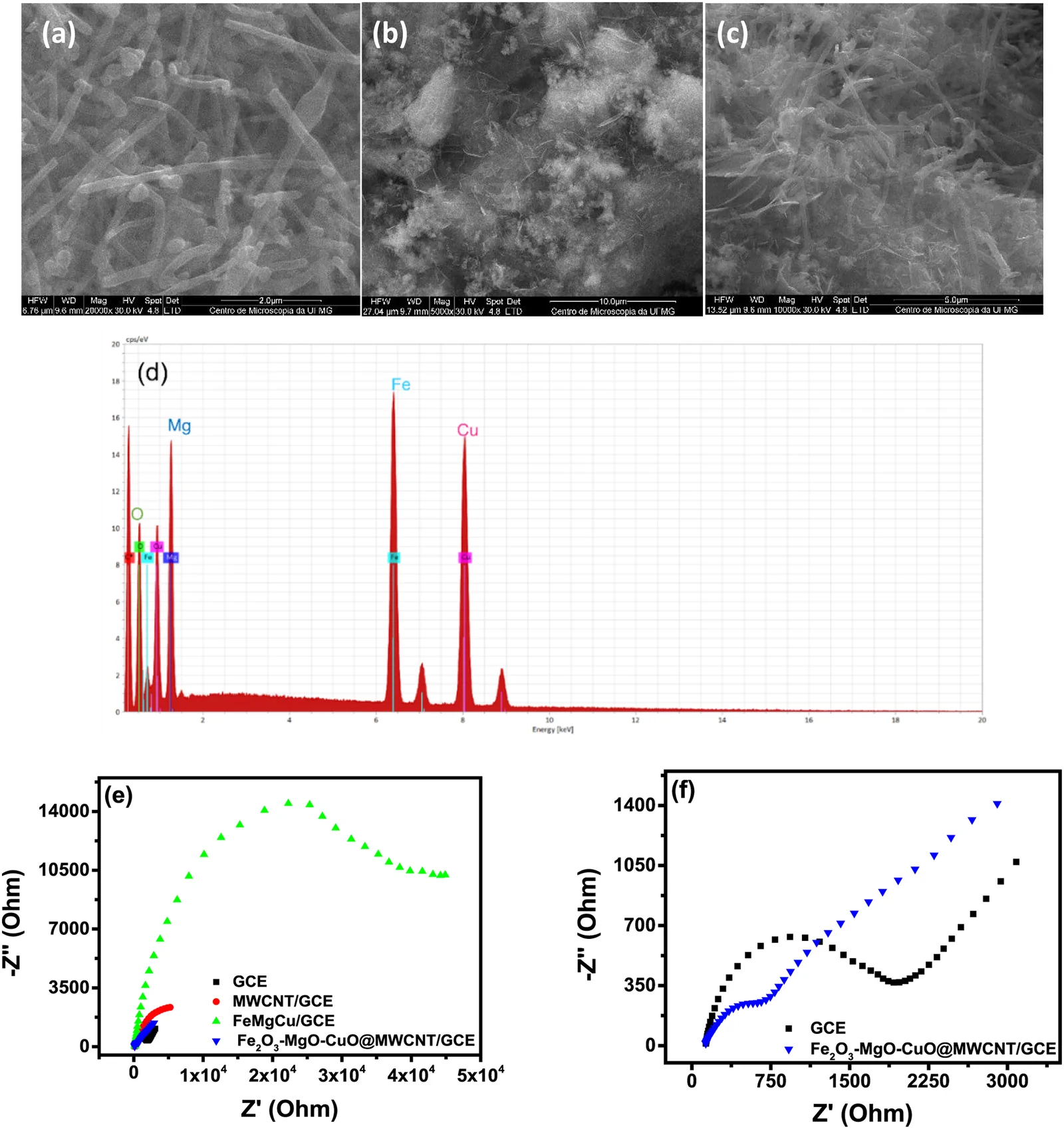
SEM images of (a) MWCNTs, (b) Fe_2_O_3_–MgO–CuO, and (c) Fe_2_O_3_–MgO–CuO/MWCNT. (d) EDS spectrum of the hybrid material. (e) Nyquist plots (EIS) for GCE, MWCNT/GCE, Fe_2_O_3_–MgO–CuO/GCE, and Fe_2_O_3_–MgO–CuO@MWCNT/GCE in 5 mmol L^–1^ K_3_[Fe­(CN)_6_] and 0.1 mol L^–1^ KCl (10^5^–0.1 Hz). (f) Enlarged view comparing bare GCE and Fe_2_O_3_–MgO–CuO@MWCNT/GCE.

For the hybrid material (Figure 2c), a more complex and hierarchical morphology is observed, in which metal oxide particles are well dispersed and anchored along the nanotube surface. This configuration reduces particle agglomeration and increases surface roughness and active area, while maintaining the conductive network provided by the MWCNTs. Such structural features are expected to be advantageous for electrochemical applications, as they can promote efficient charge transfer and increase the density of active sites.

The chemical composition was further confirmed by energy-dispersive X-ray spectroscopy (EDS) (Figure 2d), revealing the presence of C, O, Mg, Fe, and Cu, consistent with the expected composition of the hybrid material. These results corroborate the successful incorporation of the metal oxides into the MWCNT matrix.

To further evaluate this interfacial behavior, electrochemical impedance spectroscopy (EIS) was used to investigate the interfacial properties of the different electrodes. The Nyquist plots (Figure 2e) exhibit a semicircle at high and medium frequencies, associated with charge transfer. A magnified view of the high-frequency region (Figure 2f) highlights the differences in semicircle diameters among the investigated electrodes. Since the diameter of the semicircle is directly related to the charge-transfer resistance (R_ct_), the significantly smaller semicircle observed for the Fe_2_O_3_–MgO–CuO@MWCNT/GCE electrode indicates a lower interfacial charge-transfer resistance and faster electron-transfer kinetics compared to the other electrode configurations. This behavior suggests that the integration of the trimetallic oxide composite with MWCNTs facilitates charge transport at the electrode/electrolyte interface, contributing to the improved electrochemical performance of the proposed sensing platform.^38^

The Nyquist spectra were simulated by using an equivalent circuit based on the solution resistance (R_s_) in series with a parallel association of a resistor (R_ct_, charge transfer resistance), and a constant phase element (Y_0_). The R_s_ values ranged from 116 to 152 Ω across all systems, indicating minor differences in the electrolyte resistance. In contrast, substantial variations were observed in R_ct_, reflecting differences in the interfacial electron-transfer kinetics. The Fe_2_O_3_–MgO–CuO-modified electrode exhibited the highest R_ct_ (41.251 kΩ), considerably greater than that of the bare GCE (1.764 kΩ), indicating slower charge-transfer kinetics consistent with the relatively low electronic conductivity of mixed metal oxides. The MWCNT-modified electrode displayed an intermediate R_ct_ value (9.090 kΩ), whereas the Fe_2_O_3_–MgO–CuO@MWCNT/GCE hybrid exhibited the lowest R_ct_ (0.899 kΩ). This pronounced decrease demonstrates that the incorporation of MWCNTs provides efficient conductive pathways that facilitate interfacial electron transfer, thereby overcoming the relatively low electronic conductivity of the oxide phase. These results highlight the complementary contribution of the conductive MWCNT network to the mixed oxide phase. The corresponding fitting parameters are summarized in Table S1.

The heterogeneous electron transfer rate constant (k^0^) was estimated using (eq 1):^39^

1 $$k^{0}=RT/\left(n^{2}F^{2}ACRct\right)$$

where R = 8.314 J mol^–1^ K^–1^, T = 298 K, F = 96485 C mol^–1^, n = 1, A = 0.071 cm^2^, and C = 5 × 10^–6^ mol cm^–3^.

The calculated k^0^ values (Table S1) follow the same trend as R_ct_, with the hybrid electrode exhibiting the highest electron transfer rate, indicating enhanced charge-transfer kinetics resulting from the formation of the hybrid material. These results support a complementary contribution of the conductive MWCNT network to the metal oxide phase, resulting in enhanced charge-transfer kinetics. This improved interfacial behavior is consistent with the superior electrochemical performance observed for the hybrid electrode and is expected to contribute to improved sensitivity and lower detection limits in l-CYS determination.

### Electrochemical Behavior of l-Cysteine at Bare and Modified Electrodes

The electrochemical behavior of l-CYS was investigated using different electrode systems. The obtained voltammetric responses revealed significant differences in peak potentials and electrochemical activity, highlighting the influence of the electrode surface on the redox process.

For the bare GCE (Figure 3a), no anodic peak was observed in the blank solution, while a cathodic signal at approximately −0.50 V vs Ag/AgCl (sat. KCl) was detected, corresponding to the reduction of dissolved oxygen. Upon addition of l-CYS, an anodic peak appeared at ∼0.70 V vs Ag/AgCl (sat. KCl), indicating that the oxidation of the analyte requires a relatively high overpotential at the unmodified electrode, which is characteristic of sluggish electron-transfer kinetics.

**Figure 3:**
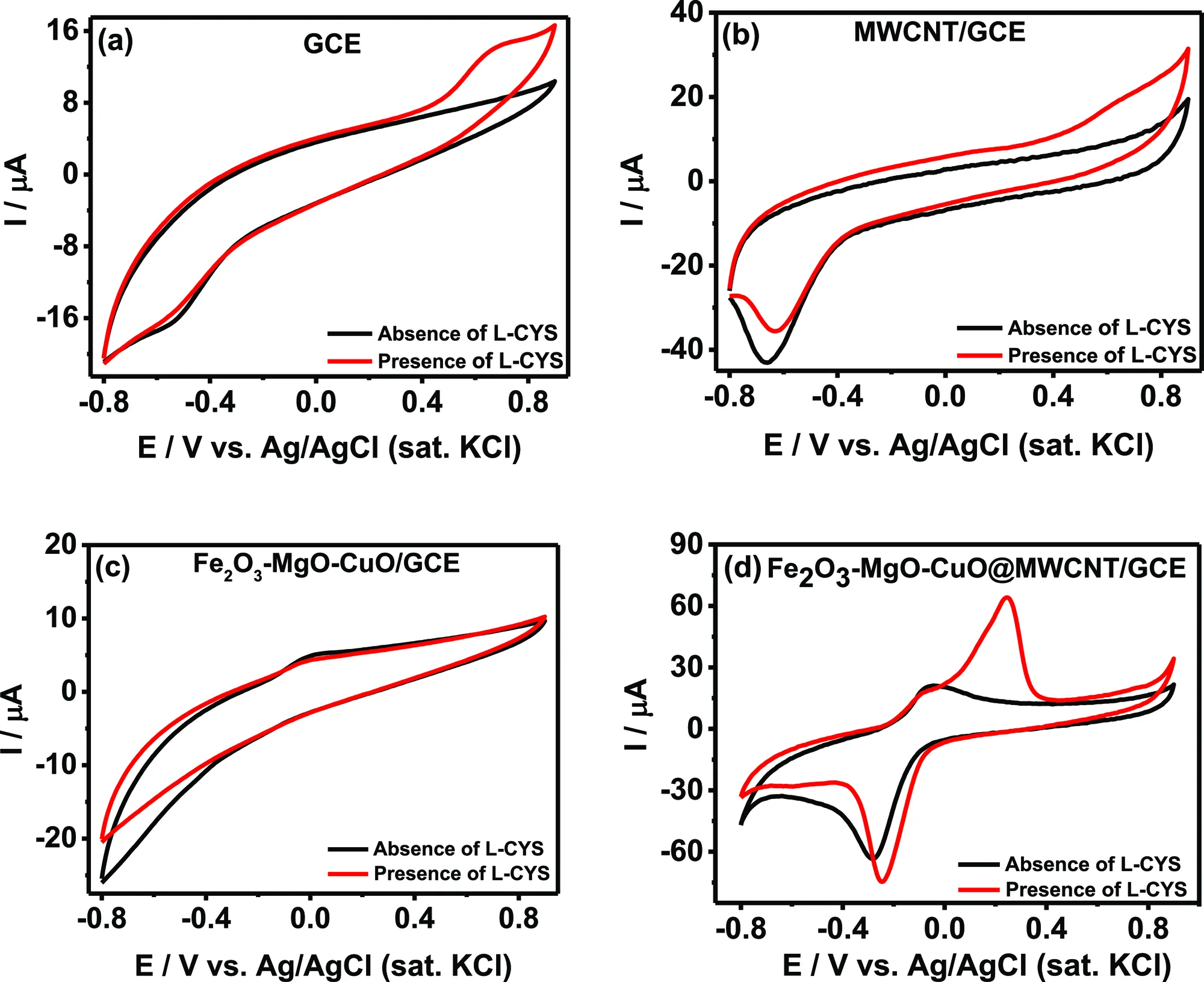
Cyclic voltammograms of (a) GCE, (b) MWCNT/GCE, (c) Fe_2_O_3_–MgO–CuO/GCE, and (d) Fe_2_O_3_–MgO–CuO@MWCNT/GCE electrodes recorded in 0.1 mol L^–1^ PBS (pH 7.0) at a scan rate of 50 mV s^–1^, in the absence and presence of l-CYS ([l-CYS] = 1000 μmol L^–1^).

A similar behavior was observed for the MWCNT/GCE (Figure 3b), where the oxidation peak of l-CYS remained at approximately ∼0.60 V vs Ag/AgCl (sat. KCl). Although MWCNTs are known to improve electrical conductivity, the absence of a significant shift in the oxidation potential indicates a limited electrocatalytic effect under these conditions. This indicates that improved conductivity alone is not sufficient to significantly reduce the overpotential for oxidation of l-CYS.

The Fe_2_O_3_–MgO–CuO/GCE electrode (Figure 3c) exhibited a negligible electrochemical response toward l-CYS, indicating that the metal oxide composite alone does not provide sufficient conductivity or accessible active sites to promote efficient electron transfer. This observation is consistent with the high charge-transfer resistance obtained from EIS analysis, confirming the intrinsically resistive nature of the material.

A markedly different response was observed for the Fe_2_O_3_–MgO–CuO@MWCNT/GCE electrode (Figure 3d). In the absence of l-CYS, anodic and cathodic peaks were observed at −0.045 V and −0.280 V vs Ag/AgCl (sat. KCl), respectively. These redox peaks can be associated with the electrochemical activity of surface Cu^2+^/Cu^+^ and Fe^3+^/Fe^2+^ redox couples present in the trimetallic oxide composite, which act as the electroactive centers responsible for the reversible electron-transfer processes observed in the voltammogram.

This assignment is further supported by cyclic voltammograms of the individual oxide-modified electrodes presented in the Supporting Information (Figure S1), where only the CuO@MWCNT/GCE and Fe_2_O_3_@MWCNT/GCE electrodes exhibit well-defined redox features, whereas MgO@MWCNT/GCE shows no significant faradaic response under the same experimental conditions.

In addition, the cyclic voltammogram obtained for Fe_2_O_3_–MgO–CuO@MWCNT/GCE electrode in the presence of l-CYS (Figure 3d) presented an oxidation peak for the analyte at a significantly lower potential (+0.25 V vs Ag/AgCl (sat. KCl)) in comparison to the other electrodes, indicating a substantial decrease in overpotential and enhanced electrocatalytic activity.

Furthermore, the increase in cathodic peak current, together with the shift in cathodic peak potential observed in the presence of l-CYS, suggests that the analyte modifies the electrochemical response of the redox-active surface species. This behavior is consistent with a possible interaction between l-CYS and electroactive surface sites associated with the Cu- and/or Fe-containing oxide phases. However, the electrochemical data obtained in the present study does not confirm the specific nature of such interaction or the formation of a surface complex.

Nevertheless, previous studies have reported interactions between thiol-containing compounds and copper species, suggesting that Cu-containing surface sites may contribute to the electrochemical response of thiol compounds.^21,40,41^ Based on these reports and on the electrochemical behavior observed in the present study, the following reduction mechanism is proposed for the cathodic detection of l-CYS on modified electrode: (1) Cu­(II) + 2RS → Cu­(II)­(RS)_2_; (2) Cu­(II)­(RS)_2_ + 2e^–^ → Cu + 2RS, where Cu­(II) represents the copper­(II) ion from CuO and RS represents the thiol. However, this possible mechanism is only a proposition since here is not presented a detailed investigation about the interaction of l-CYS with the metal oxides.

Overall, these findings demonstrate that electrode modification with the Fe_2_O_3_–MgO–CuO@MWCNT nanocomposite is essential for the efficient electrochemical response toward l-CYS. The metal oxides are likely to provide the electroactive sites responsible for the electrocatalytic process, whereas the MWCNT network establishes an efficient conductive pathway that promotes rapid electron transfer. The absence of a significant response for the electrode containing only the metal oxides (without MWCNTs) further demonstrates that electrical conductivity is a limiting factor. Therefore, the role of MWCNTs extends beyond that of simple support, being essential for establishing effective electronic communication between the electroactive metal centers and the electrode surface, thereby enabling the enhanced analytical performance observed for l-CYS detection.

Further insight into this behavior is provided by the control experiments presented in Supporting Information (Figure S1). The individual oxide-modified electrodes exhibited distinct electrochemical responses toward l-CYS, with CuO@MWCNT/GCE showing an anodic peak at about +0.25 V and a small shift and increase of the cathodic peak at about −0.25 V while the Fe_2_O_3_@MWCNT/GCE displaying a better-defined reduction peak without a significant increase in cathodic current. Likewise, the Fe_2_O_3_–CuO@MWCNT/GCE binary composite exhibited a more defined cathodic response and the characteristic oxidation peak of l-CYS. However, both the anodic and cathodic current responses remained considerably lower than those observed for the Fe_2_O_3_–MgO–CuO@MWCNT/GCE electrode. Although MgO@MWCNT/GCE exhibited no significant faradaic response toward l-CYS under the investigated conditions, its incorporation into the Fe_2_O_3_–CuO binary system was associated with a substantial increase in the electrochemically accessible surface area (Figure S2). The electrochemically active surface area increased from 0.003 cm^2^ for Fe_2_O_3_–CuO@MWCNT/GCE to 0.043 cm^2^ for Fe_2_O_3_–MgO–CuO@MWCNT/GCE, corresponding to an approximately 14-fold increase. This result indicates that incorporation of MgO into the Fe_2_O_3_–CuO@MWCNT architecture contributes to an increase in the electrochemically accessible surface area. Moreover, the enhanced electrochemical response observed for Fe_2_O_3_–MgO–CuO@MWCNT/GCE was not reproduced by the individual MgO-based system or by the Fe_2_O_3_–CuO binary system alone under the investigated conditions. These findings indicate that the enhanced response is associated with the overall trimetallic oxide/MWCNT architecture rather than with the electrochemical activity of a single oxide or the Fe_2_O_3_–CuO binary system alone.

In addition, the effect of dissolved oxygen on the electrochemical response of the Fe_2_O_3_–MgO–CuO@MWCNT/GCE system for l-CYS determination was also investigated (Figure 4). Voltammograms were recorded in the absence and presence of l-CYS under oxygenated and deoxygenated conditions in 0.1 mol L^–1^ PBS (pH 7.0) at a scan rate of 100 mV s^–1^.

**Figure 4:**
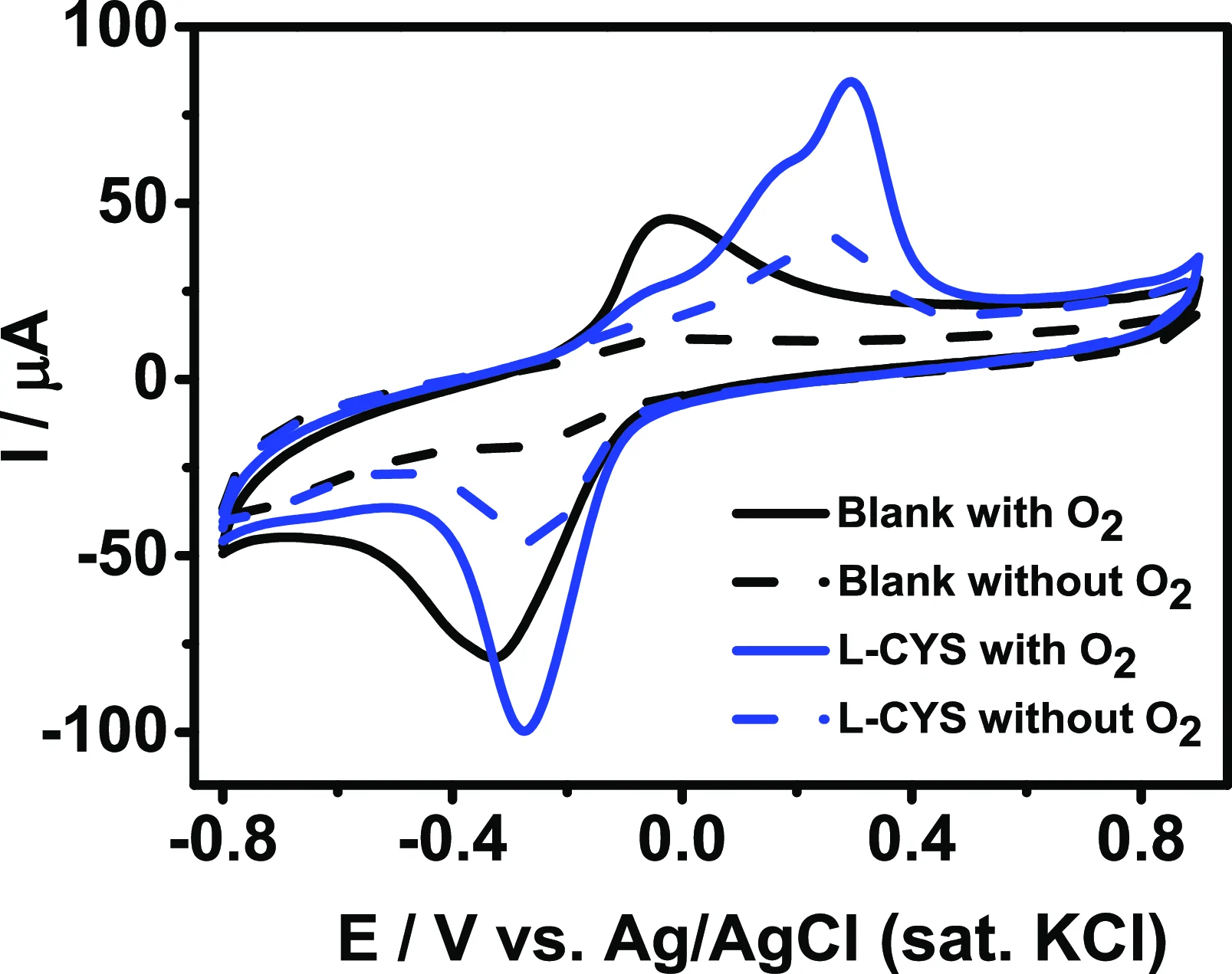
Effect of dissolved oxygen on the electrochemical response of l-CYS at the Fe_2_O_3_–MgO–CuO@MWCNT/GCE sensor in 0.1 mol L^–1^ PBS (pH 7.0) at a scan rate of 100 mV s^–1^. Voltammograms were recorded in the absence (blank) and presence of l-CYS under both oxygenated (+O_2_) and deoxygenated (−O_2_) conditions, where (+) and (−) indicate the presence and absence of dissolved oxygen, respectively. [l-CYS] = 1000 μmol L^–1^.

In the absence of l-CYS (blank solution), as observed in the black voltammograms, a well-defined redox pair characteristic of the modified electrode was obtained under aerated conditions (*E*_ap_ = −0.020 V, I_ap_ = 40.29 μA; E_cp_ = −0.33 V, I_cp_ = −56.00 μA). After oxygen removal, this redox signal remained detectable, although with a significant decrease in current intensity (I_ap_ = 6.99 μA; I_cp_ = −4.82 μA) and loss of peak definition, indicating that dissolved oxygen contributes significantly to the overall current response, particularly in the cathodic region.

In the presence of l-CYS, as observed in the blue voltammograms, a marked increase in current response was observed under both conditions. Under aerated conditions, a cathodic peak at E_cp_ = −0.27 V (I_cp_ = −79.76 μA) and an anodic peak at *E*_ap_ = 0.29 V (I_ap_ = 66.37 μA) were observed. After oxygen removal, a similar voltammetric profile was maintained, although with reduced currents (E_cp_ = −0.27 V, I_cp_ = −29.73 μA; *E*_ap_ = 0.27 V, I_ap_ = 26.63 μA), indicating that both anodic (oxidation of l-CYS) and cathodic processes remain observable under deaerated conditions.

In order to isolate the analytical contribution of l-CYS, the net cathodic current (ΔI_cp_) was estimated by subtracting the blank signal from the signal obtained in the presence of the analyte. This net current remained essentially unchanged within experimental variation between aerated (−24.49 ± 0.68 μA) and deaerated (−24.55 ± 0.34 μA) conditions (n = 3). These results indicate that, although dissolved oxygen increases the overall current magnitude and affects peak definition, it does not significantly influence the analytical signal associated with l-CYS.

The net electrochemical response of l-CYS was not significantly affected by dissolved oxygen, indicating that the analytical signal is independent of this species under the studied conditions. This eliminates the need for deaeration, simplifying the procedure and enabling faster analysis. The influence of oxygen is mainly limited to background current and peak definition. Therefore, subsequent experiments were performed under ambient conditions without deoxygenation, ensuring both simplicity and reliable analytical performance.

### Effect of pH and Supporting Electrolyte on the Electrochemical Response of l-Cysteine

The influence of pH on the voltammetric response was investigated to evaluate the effect of solution pH and establish the optimal analytical conditions. In acidic media, the addition of l-CYS led to a decrease in the current associated with the electrode modifier (data not shown); therefore, the study was focused on neutral to mildly alkaline conditions, where a well-defined and analytically useful response was obtained (Figure 5a–e).

**Figure 5:**
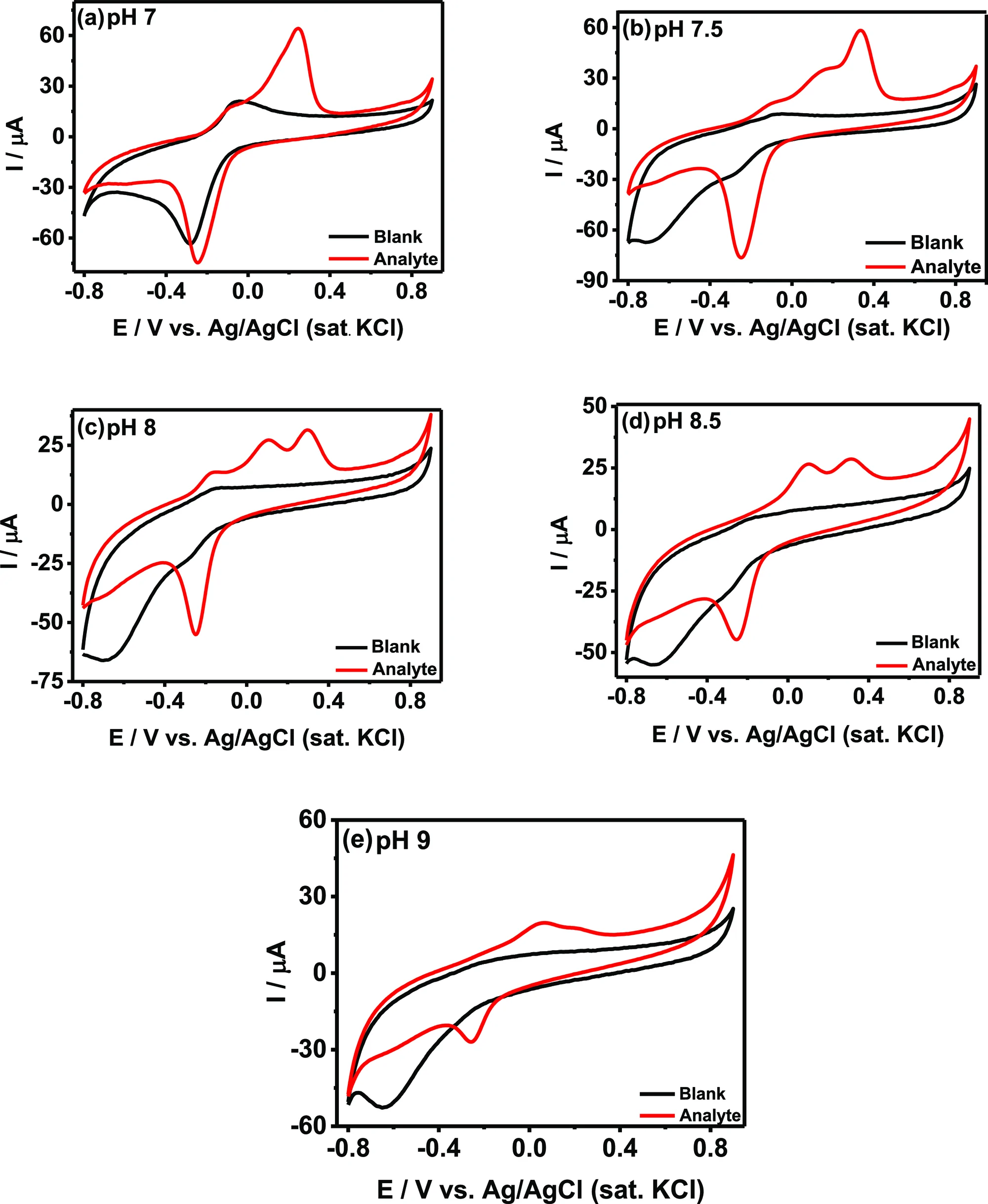
Cyclic voltammograms of l-CYS recorded at different pH values using the Fe_2_O_3_–MgO–CuO@MWCNT/GCE electrode in 0.1 mol L^–1^ PBS at a scan rate of 50 mV s^–1^: (a) pH 7.0, (b) pH 7.5, (c) pH 8.0, (d) pH 8.5, and (e) pH 9.0.

In all voltammograms, the black curves correspond to the response of the Fe_2_O_3_–MgO–CuO/MWCNT/GCE electrode in the absence of l-CYS, while the red curves represent the response in the presence of l-CYS. The voltammetric profiles recorded at different pH values (Figure 5a–e) revealed that both anodic and cathodic responses are influenced by the solution pH. Regarding the anodic response, the voltammetric profiles (Figure 5) show a clear evolution of the oxidation features with pH.

Across all investigated pH values (7.0–9.0), the voltammogram of the Fe_2_O_3_–MgO–CuO/MWCNT/GCE electrode exhibits a characteristic profile of a metal oxide-based electrocatalytic system. In the absence of cysteine, the voltammetric response is associated with redox-active Cu- and Fe-containing surface species in the trimetallic oxide composite, with efficient electron transfer facilitated by the MWCNT network. In the presence of l-CYS, a significant increase in the cathodic current is observed, indicating that the analyte enhances the electrochemical response of the system. At higher pH values, changes in current intensity may be associated with variations in the protonation state of l-CYS, which can influence the kinetics of the electrochemical process.

At pH 7.0, a well-defined anodic peak is observed at approximately +0.25 V, attributed to l-CYS oxidation, partially overlapped with a contribution from the modifier at lower potentials. At pH 7.5, the anodic region becomes more complex, with the appearance of two contributions: a less defined wave at lower potential (≈+0.16 V) and a more pronounced peak at higher potential (≈+0.34 V), indicating partial separation of oxidation processes.

At pH 8.0 and 8.5, these anodic features become more distinguishable, although still not fully resolved, suggesting that the oxidation of l-CYS may involve multiple, partially overlapping processes. At pH 9.0, the anodic response becomes less defined, with the main peak appearing less resolved and accompanied by a secondary contribution, indicating loss of resolution under more alkaline conditions. In addition, the anodic currents decrease compared to pH 7.0–7.5, indicating reduced electrochemical response at higher pH values.

The variation of the cathodic peak current shows a pronounced dependence on pH (Figure 6a), increasing significantly from −14.27 μA at pH 7.0 to a maximum value of −57.86 μA at pH 7.5. At higher pH values (8.0–9.0), a progressive decrease in current is observed, reaching −12.02 μA at pH 9.0, indicating reduced electrochemical efficiency under more alkaline conditions. As shown in Figure 6a, the maximum cathodic current is obtained at pH 7.5, while the peak potential remains essentially unchanged throughout the evaluated range (Figure 6b). This behavior suggests that pH primarily affects the kinetics and efficiency of the process rather than its fundamental electrochemical pathway.

**Figure 6:**
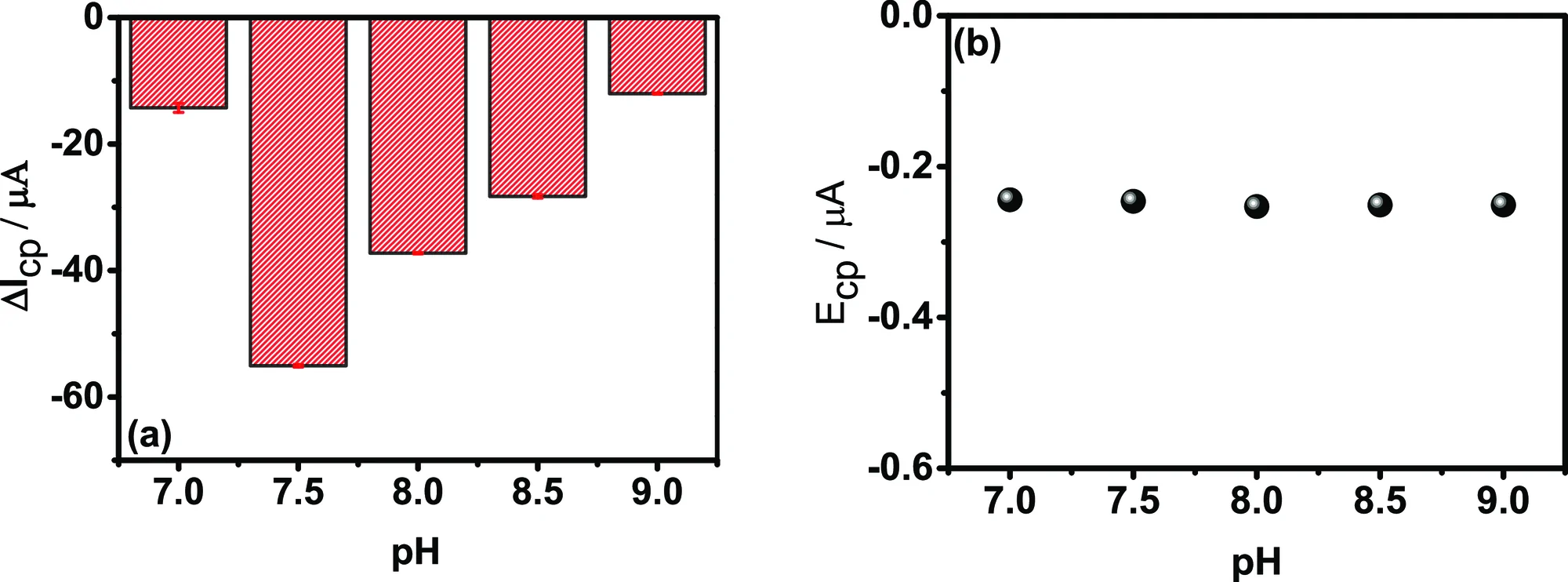
(a) Dependence of the cathodic peak current (I_cp_) of l-CYS on pH (7.0–9.0) at the Fe_2_O_3_–MgO–CuO@MWCNT/GCE electrode in PBS, obtained by cyclic voltammetry at a scan rate of 50 mV s^–1^ (*n* = 3). (b) Variation of the cathodic peak potential (E_cp_) as a function of pH. Data extracted from Figure 5.

Based on these results, pH 7.5 was selected as the optimal condition for l-CYS determination. Although the anodic response provides useful mechanistic information, it is characterized by overlapping features and reduced resolution, particularly at higher pH values, which limits its analytical applicability.

In contrast, the cathodic signal remains better defined, more stable, and less affected by overlapping processes, making it more suitable for reliable quantification. The weak dependence of the cathodic peak potential on pH suggests that the electrochemical process is not strongly governed by proton-coupled electron transfer within the investigated range. Consequently, the proposed sensor offers the practical advantage of operating under near-neutral conditions without stringent pH control, which is beneficial for routine and real-sample analysis.

Following the investigation of the pH effect, the influence of the supporting electrolyte was evaluated to identify the most suitable medium for analytical applications. The voltammetric responses obtained in different supporting electrolytes are presented in Figure 7a–d, corresponding to PBS, McIlvaine (MCV), Britton–Robinson (BR), and Tris, respectively, while the corresponding cathodic peak currents are summarized in Figure 7e. As observed, both the peak potential and current intensity are affected by the electrolyte composition, indicating that the electrochemical response is sensitive to the chemical environment of the solution.

**Figure 7:**
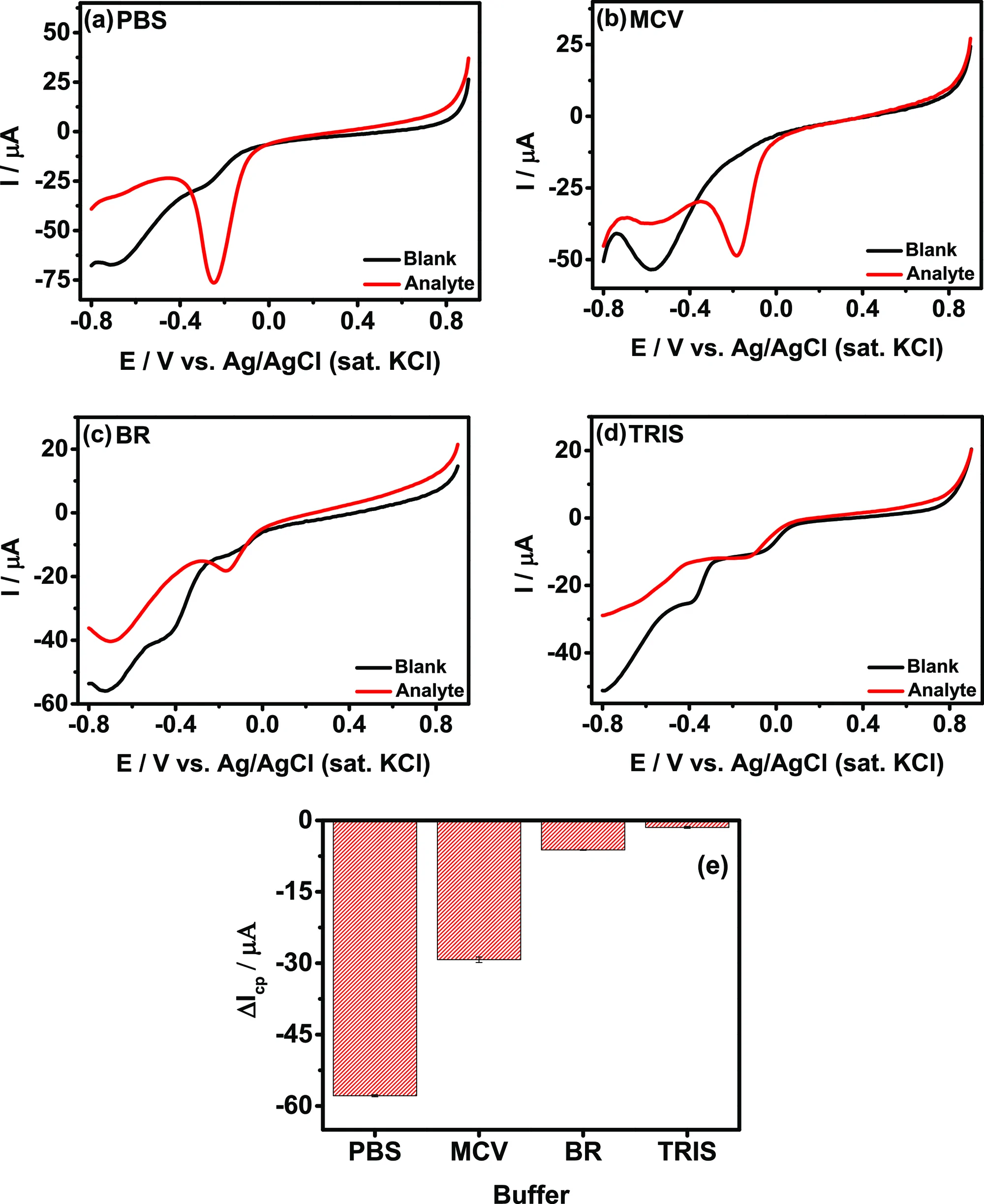
LSV voltammograms of l-CYS at the Fe_2_O_3_–MgO–CuO@MWCNT/GCE electrode in different supporting electrolytes: (a) PBS, (b) McIlvaine buffer (MCV), (c) Britton–Robinson buffer (BR), and (d) Tris buffer, recorded at a scan rate of 50 mV s^–1^; [buffers] = 0.1 mol L^–1^. (e) Comparison of the cathodic peak currents obtained in each medium (*n* = 3).

Although cathodic peaks were observed in all evaluated media, marked differences in current intensity were evident. Among the tested buffers, PBS exhibited the highest cathodic peak current, indicating more favorable conditions for the electrochemical process. This behavior can be associated with improved solution conductivity and a more stable interfacial environment in phosphate buffer, which facilitates charge transfer.

In contrast, lower current responses were obtained in MCV, BR, and Tris media, suggesting lower efficiency of the electrochemical response under these conditions. These differences may be related to variations in ionic strength, buffer composition, and interactions at the electrode/solution interface, which can influence the overall kinetics of the process.

The dependence of the electrochemical response on the supporting electrolyte composition can be discussed considering the general characteristics of each buffer system. PBS provides a relatively stable and reproducible medium, favoring consistent electrochemical signals. On the other hand, more complex buffer systems such as McIlvaine and Britton–Robinson may introduce additional interactions in solution, potentially affecting the electrochemical response. Similarly, Tris buffer may alter the interfacial environment due to its chemical nature, leading to lower current responses compared to PBS. It is important to note that, although clear differences among electrolytes are observed, specific interactions between buffer components and the electrode surface cannot be unequivocally determined based solely on voltammetric data. Based on these results, PBS was selected as the supporting electrolyte for l-CYS determination, as it provides the highest cathodic current among the evaluated media, resulting in improved analytical sensitivity.

### Effect of Scan Rate on the Electrochemical Behavior of l-Cysteine

The effect of scan rate on the electrochemical behavior of the proposed sensor was investigated by linear sweep voltammetry (LSV) in the absence and presence of l-CYS (Figure 8). In the absence of l-CYS (Figure 8a), the cathodic response becomes less defined with increasing scan rate, suggesting kinetic limitations associated with the redox-active sites of the electrode modifier. In contrast, in the presence of l-CYS (Figure 8b), a well-defined cathodic peak is observed over the entire scan rate range (10–100 mV s^–1^), with peak current increasing systematically, indicating a stable electrochemical response.

**Figure 8:**
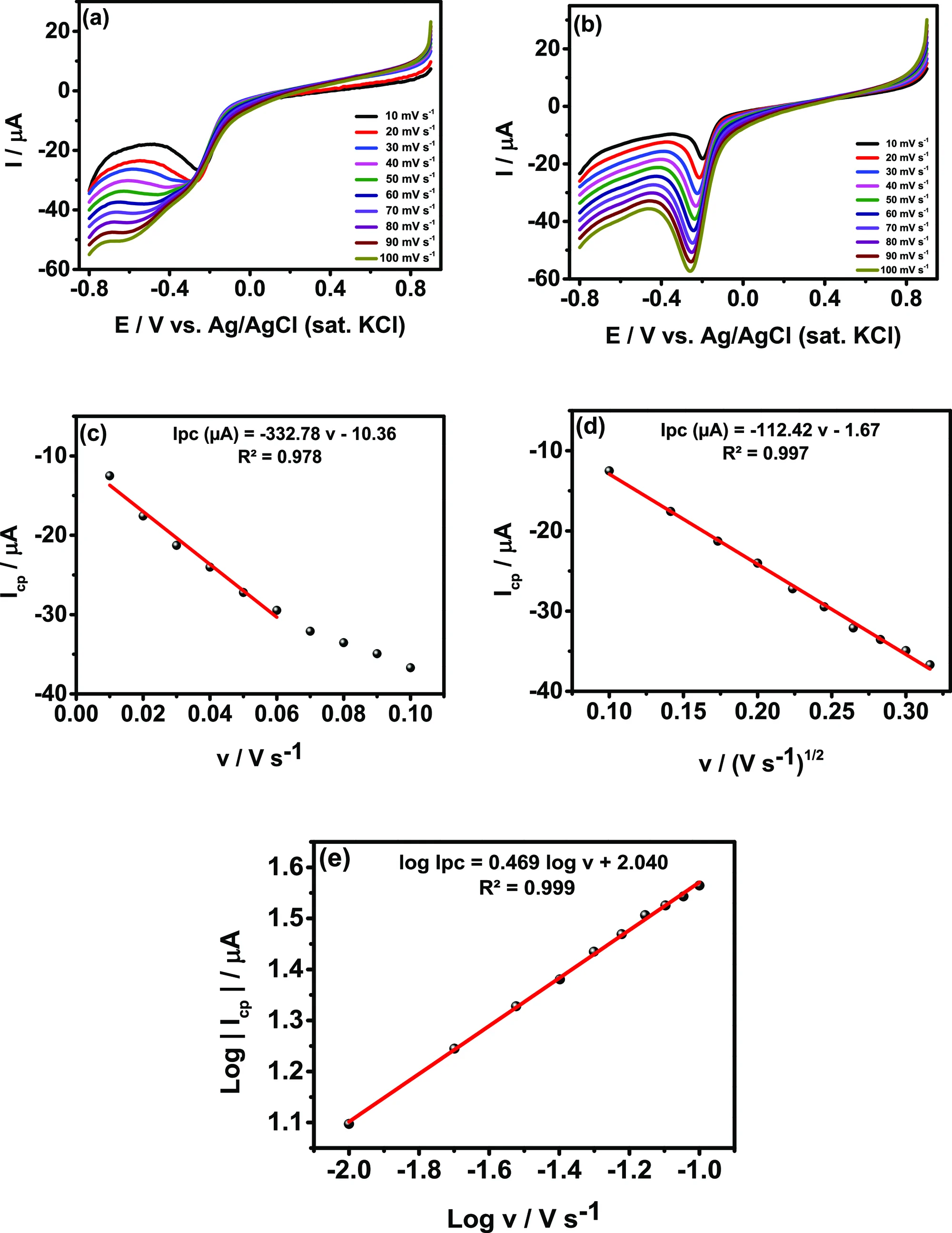
Effect of scan rate (10–100 mV s^–1^) on the electrochemical response of the Fe_2_O_3_–MgO–CuO@MWCNT/GCE electrode in 0.1 mol L^–1^ PBS (pH 7.5): (a) linear sweep voltammograms recorded in the absence of l-CYS; (b) linear sweep voltammograms recorded in the presence of l-CYS; (c) plot of cathodic peak current (I_cp_) versus scan rate (ν); (d) plot of I_cp_ versus the square root of scan rate (ν^1/2^); and (e) log–log plot of I_cp_ versus ν.

The dependence of cathodic peak current on scan rate was analyzed to elucidate the controlling mechanism. A linear relationship between peak current and scan rate (ν) was observed at lower scan rates (10–60 mV s^–1^) (Figure 8c), indicating a contribution from surface-controlled processes. However, over the full range (10–100 mV s^–1^), the peak current varies linearly with the square root of scan rate (ν^1/2^) (Figure 8d), characteristic of diffusion-controlled behavior. This is further supported by the log–log plot (Figure 8e), which yielded a slope of 0.46, close to the theoretical value of 0.50 for diffusion-controlled systems.

These results indicate that, although surface processes contribute at lower scan rates, the electrochemical response in the presence of l-CYS is predominantly diffusion-controlled.

### Electrochemical Detection of l-CYS at the Fe_2_O_3_–MgO–CuO@MWCNT/GCE Sensor

The calibration curve for l-CYS was obtained by linear sweep voltammetry under optimized conditions over a concentration range of 0.5–1000 μmol L^–1^ (Figure 9a). The analytical response was evaluated by plotting cathodic peak current against of the logarithm of the l-CYS concentration (Figure 9b).

**Figure 9:**
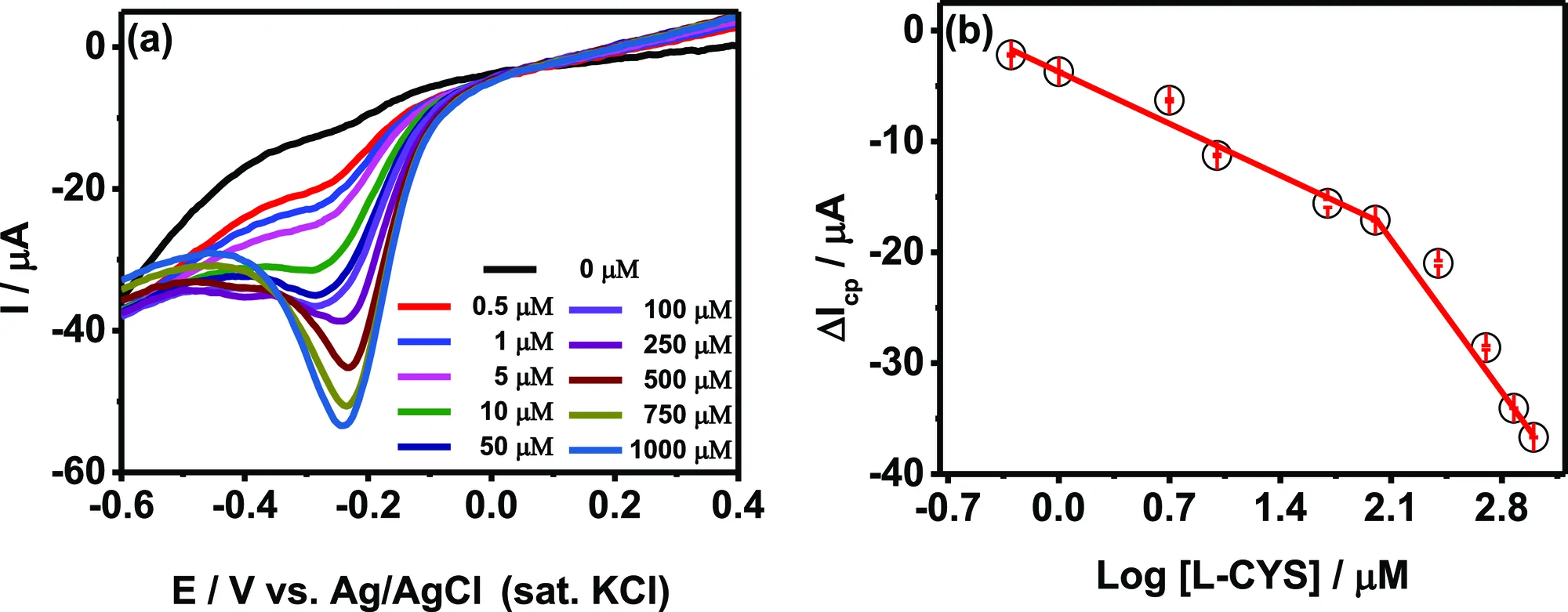
(a) Linear sweep voltammograms recorded from positive to negative potentials for successive additions of l-CYS over the concentration range of 0.5–1000 μmol L^–1^ in 0.1 mol L^–1^ PBS (pH 7.5) at a scan rate of 50 mV s^–1^; (b) corresponding calibration plot of cathodic peak current (I_cp_) as a function of log [l-CYS] (μmol L^–1^) (*n* = 3).

The calibration plot exhibited two linear regions. In the range of 0.5–100 μmol L^–1^, the cathodic peak current followed the relationship I (μA) = −3.72 – 6.68 log C (R^2^ = 0.992), while in the range of 100–1000 μmol L^–1^, it followed I (μA) = 22.34 – 19.63 log C (R^2^ = 0.996). Both regions showed excellent linearity, indicating reliable analytical performance over a wide concentration range. The presence of two linear regions suggests different electrochemical regimes, likely related to changes in surface coverage and mass transport. The lowest detectable concentration of l-CYS with the Fe_2_O_3_–MgO–CuO@MWCNT/GCE electrode was 0.5 μmol L^–1^.

To further assess the analytical performance of the proposed sensor, a comparison with previously reported methods for l-CYS determination was carried out (Table 1).^4,5,9,11,12,42–48^ A comparison with previously reported methods reveals that the proposed sensor exhibits competitive analytical performance, particularly in terms of detection limit and operational simplicity.

**Table 1:** Comparison of the Proposed Sensor with Previously Reported Methods for l-CYS Determination^a^

Method/material	Technique	Linear range (μmol L^–1^)	LOD (μmol L^–1^)	Ref
Chromatography	HPLC	0.15–500	0.04	^4^
Chromatography	HPLC-UV	1–20	-	^5^
Colorimetric (Mn-TA platform)	Colorimetry	8.26–90.86	2.28	^9^
Electrochemical (Ag@rGO/GCE)	Amperometry	0.1–470	0.057	^11^
Electrochemical (ZnO/Co_3_O_4_/rGO/GCE)	Amperometry	20–800	0.01	^12^
Electrochemical (Ni@AC/GCE)	CV	0.8–8	0.15	^42^
		8–85		
Electrochemical (PMo_9_V_3_/CoTsPc/ITO)	Amperometry	0.25–170	0.10	^43^
		170–3950		
Electrochemical (BiPr/GCE)	CV	1–2000	0.21	^44^
Electrochemical (Co–La oxides/rGO)	CV	1–888	0.10	^45^
Electrochemical RAILs/CB/GCE	CV	0.05–80	0.04	^46^
Eletrochemical (Au NDs@FCF)	Amperometry	0.1–3	0.00016	^47^
	LSV	10–300	1.1	
Electrochemical (MXene@PDA/MOF)	VPD	0.01–5	0.00374	^48^
Electrochemical (Fe_2_O_3_–MgO–CuO@MWCNT/GCE)	LSV	0.5–100	0.5	This work
		100–1000		

a HPLC: high-performance liquid chromatography; HPLC-UV: high-performance liquid chromatography with ultraviolet detection; Mn-TA: manganese–tannic acid; Ni@AC: nickel nanoparticles supported on activated carbon; PMo_9_V_3_: phosphomolybdovanadate polyoxometalate; CoTsPc: cobalt tetrasulfonated phthalocyanine; BiPr: bismuth purple; Co–La oxides: cobalt–lanthanum oxides; RAILs: room-temperature amine ionic liquids; CB: carbon black; Au NDs: gold nanodendrites; FCF: functionalized carbon felt; MXene: two-dimensional transition metal carbides and/or nitrides; PDA: polydopamine; MOF: metal–organic framework.

Electrochemical sensors reported in the literature show varying performance depending on the electrode material. Although some systems achieve low detection limits, they often involve more complex synthesis procedures or exhibit narrower linear ranges. In contrast, the Fe_2_O_3_–MgO–CuO@MWCNT/GCE sensor combines a wide dynamic range and it is able to detect l-CYS from cathodic scans while a high number of electrochemical devices are able to detect the analyte from its oxidation. These features highlight the advantage of the proposed platform as a cost-effective and efficient alternative for l-CYS determination, particularly for applications requiring rapid and sensitive analysis in complex matrices.

### Selectivity, Repeatability, and Reproducibility

The influence of potential interfering species was evaluated by monitoring their effect on the cathodic peak current of l-CYS (500 μmol L^–1^) at a 1:1 concentration ratio. As shown in Table S2, most interferents did not significantly affect the electrochemical response, demonstrating the good selectivity of the sensor. Tryptophan exhibited a more pronounced effect, likely due to its intrinsic electroactivity; however, the observed variation does not compromise the overall analytical performance. The fact that the change observed in the current responses obtained after the addition of interfering species remained below 10% indicates that the Fe_2_O_3_–MgO–CuO@MWCNT/GCE electrode exhibits good selectivity toward the target analyte and is resistant to interference effects.

Repeatability (n = 10) and reproducibility (n = 4) of the Fe_2_O_3_–MgO–CuO@MWCNT/GCE sensor were evaluated using a 500 μmol L^–1^ l-CYS solution. The corresponding linear sweep voltammograms are presented in Figure 10a and c, demonstrating the high consistency of the electrochemical responses obtained with the same electrode and with independently prepared electrodes, respectively. The statistical evaluation of the peak currents is summarized in Figure 10b and d, yielding relative standard deviation (RSD) values of 1.58% for repeatability and 1.82% for reproducibility, confirming the excellent precision and reliability of the proposed sensor. The response observed over ten consecutive measurements using the same electrode additionally demonstrates good short-term operational stability, indicating that the sensor maintained its analytical response during repeated use. Overall, these results demonstrate good precision, reproducibility, and short-term operational robustness of the proposed sensing platform.

**Figure 10:**
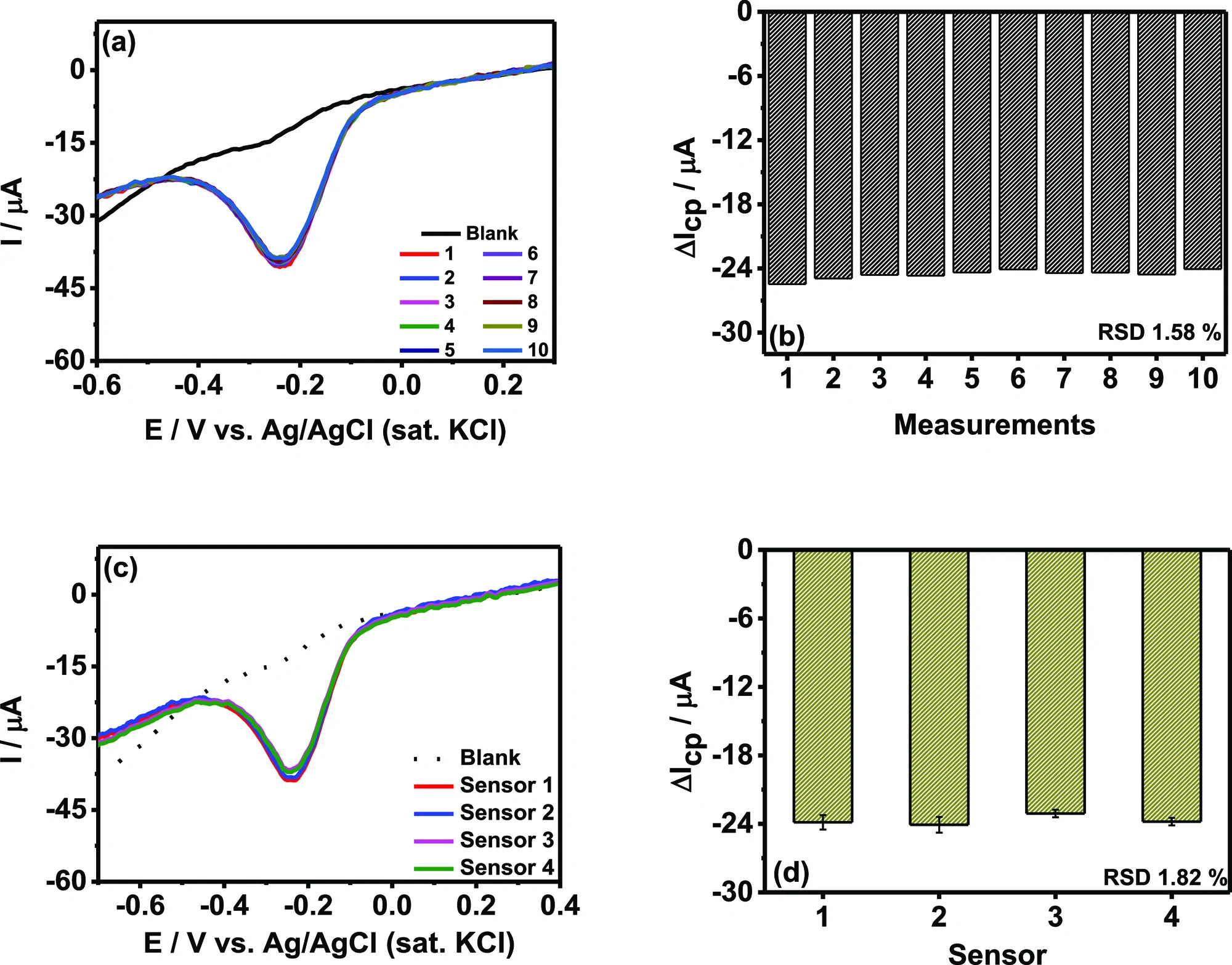
Precision evaluation of the Fe_2_O_3_–MgO–CuO@MWCNT/GCE sensor for l-CYS determination: (a) linear sweep voltammograms obtained in the repeatability study (*n* = 10); (b) corresponding cathodic peak currents (I_cp_); (c) linear sweep voltammograms obtained in the reproducibility study (*n* = 4); and (d) corresponding cathodic peak currents (I_cp_).

### Application of the Sensor in Human Urine and Breast Milk Samples

The applicability of the proposed electrochemical sensor was evaluated for the determination of l-CYS in human urine and breast milk samples using the standard addition method. The obtained results are summarized in Table 2.

**Table 2:** Recovery Study for the Determination of l-CYS in Human Urine and Breast Milk Samples (n = 3)

Sample	Added concentration (μmol L^–1^)	Found concentration (μmol L^–1^)	Recovery (%)
Human urine	0	ND^a^	-
	50	51.54 (±0.067)	103.08
	100	96.43 (±0.453)	96.43
Breast milk	0	ND^a^	-
	50	51.59 (±0.316)	103.18
	100	97.61 (±0.189)	97.61

a ND = not detected.

After spiking, the urine samples showed recoveries of 103.08% and 96.43% at concentration levels of 50 and 100 μmol L^–1^, respectively, while the breast milk samples exhibited recoveries of 103.18% and 97.61%. The corresponding concentration values are expressed as mean ± standard deviation (n = 3). No endogenous l-CYS was detected in the samples analyzed under the experimental conditions employed. This observation may be associated with the low concentration of free l-CYS in the analyzed samples and the sample preparation procedure adopted prior to analysis, including the dilution step. Nevertheless, the satisfactory recoveries obtained using the standard addition method support the preliminary applicability of the proposed sensor to these complex biological matrices. The cathodic response observed at low potential provides an attractive analytical feature for l-CYS determination. However, these recovery results demonstrate the preliminary applicability of the proposed sensor rather than providing independent validation of analytical accuracy, since the proposed sensor was not compared with an established chromatographic method, such as HPLC or LC-MS/MS, in the present study.

## Conclusion

In summary, a Nafion-modified Fe_2_O_3_–MgO–CuO@MWCNT nanocomposite was successfully developed as an electrochemical sensing platform for l-CYS determination. The combination of the trimetallic oxide composite and MWCNTs resulted in enhanced electron-transfer kinetics and electrocatalytic activity, enabling l-CYS detection at a low operating potential with a low detection limit, two wide linear ranges, and excellent selectivity, repeatability, and reproducibility. The proposed sensor was successfully applied to the determination of l-CYS in human urine and breast milk samples, providing satisfactory recovery values and supporting its preliminary applicability to complex biological matrices. Overall, these findings highlight the potential of the proposed nanocomposite as a robust and sensitive platform for electrochemical sensing. Future studies may focus on expanding the application of the proposed sensing platform to diverse real matrices and exploring the Fe_2_O_3_–MgO–CuO trimetallic oxide as a promising electrocatalytic material for the development of new electrochemical sensing platforms for different target analytes.

## Supplementary Material



## Data Availability

All data of this study are available within the paper and its Supporting Information.

## Notes

Charge transfer resistance (R_ct_) and heterogeneous electron transfer rate constant (k^0^) for different electrode systems; effect of potentially interfering species on the determination of l-CYS; cyclic voltammograms recorded for CuO@MWCNT/GCE, Fe_2_O_3_@MWCNT/GCE, MgO@MWCNT/GCE, and Fe_2_O_3_–CuO@MWCNT/GCE; electroactive surface areas of Fe_2_O_3_–CuO@MWCNT/GCE and Fe_2_O_3_–MgO–CuO@MWCNT/GCE determined using the Randles–Ševčík equation (PDF)
