# Supplementary material for: Fe2O3–MgO–CuO@MWCNT Nanocomposite for Highly Sensitive Electrochemical Detection of l‑Cysteine at Low Potential in Biological Samples
Source: ACS Omega. 2026 Oct 14;11(42):64840–55. doi: 10.1021/acsomega.6c05509 (PMC13717787; doi:10.1021/acsomega.6c05509)
Supplement: Supplementary file 1 [file ao6c05509_si_001.pdf]

# Supporting Information (for publication)

# $Fe_2O_3$–MgO–CuO@MWCNT Nanocomposite for Highly Sensitive Electrochemical Detection of L-Cysteine at Low Potential in Biological Samples

*Danilo B. Ribeiro,*[1] *Carolina A. Carvalho,*[1] *Natilene M. Brito,*[2] *Flavio S. Damos* [1*] *and Rita C. S. Luz* [1*]

[1]*Universidade Federal do Maranhão, Avenida dos Portugueses, Nº 1966, Bacanga, CEP 65080-805, São Luís-MA, Brasil*

[2]*Instituto Federal de Educação, Ciência e Tecnologia do Maranhão, Avenida Getúlio Vargas, Nº 04, Monte Castelo, CEP 65030-005, São Luís-MA, Brasil*

**Table S1.** Charge transfer resistance (Rct) and heterogeneous electron transfer rate constant ($k^0$) for different electrode systems, obtained from electrochemical impedance spectroscopy measurements.

| Electrode System | Rct (Ω) | $k^0$ (cm $s^{-1}$) |
|---|---|---|
| **Bare GCE** | 1764 | 4.25 x $10^{-4}$ |
| **MWCNT/GCE** | 9090 | 8.25 x $10^{-5}$ |
| **$Fe_2O_3$–MgO–CuO/GCE** | 41251 | 1.82 x $10^{-5}$ |
| **$Fe_2O_3$–MgO–CuO@MWCNT/GCE** | 899 | 8.34 x $10^{-4}$ |

**Table S2.** Evaluation of the effect of potential interfering species on the determination of L-CYS.

| Interferents | RSD % |
|---|---|
| **Uric acid** | 1.12 |
| **Citric acid** | 1.62 |
| **Glucose** | 4.57 |
| **Reduced glutathione** | 3.26 |
| **Tryptophan** | 9.68 |
| **Urea** | 4.51 |

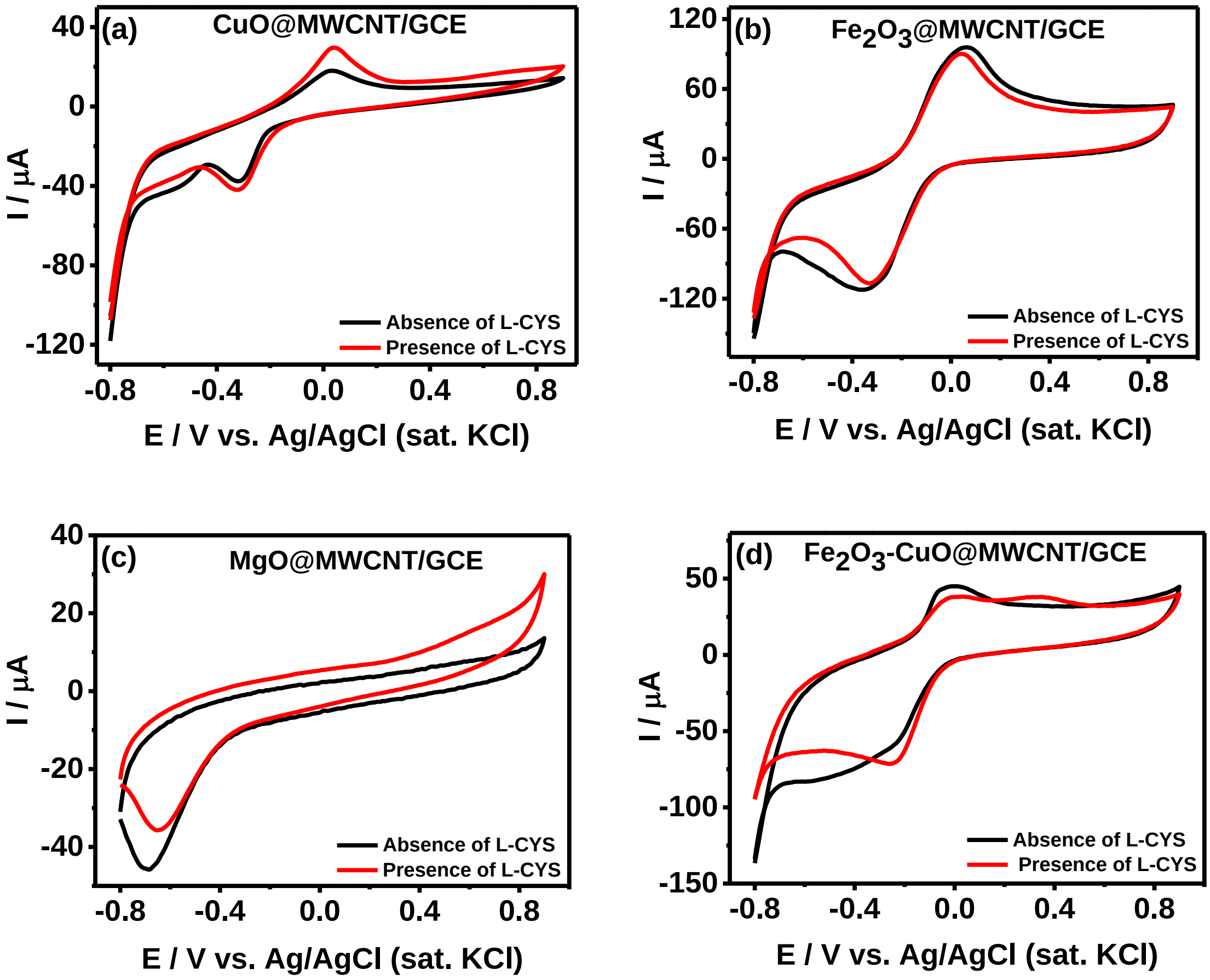


**Figure S1.** Cyclic voltammograms recorded in 0.1 mol $L^{-1}$ PBS (pH 7.0) at a scan rate of 50 mV $s^{-1}$ in the absence and presence of L-CYS for (a) CuO@MWCNT/GCE, (b) $Fe_2O_3$@MWCNT/GCE, (c) MgO@MWCNT/GCE, and (d) $Fe_2O_3$–CuO@MWCNT/GCE.

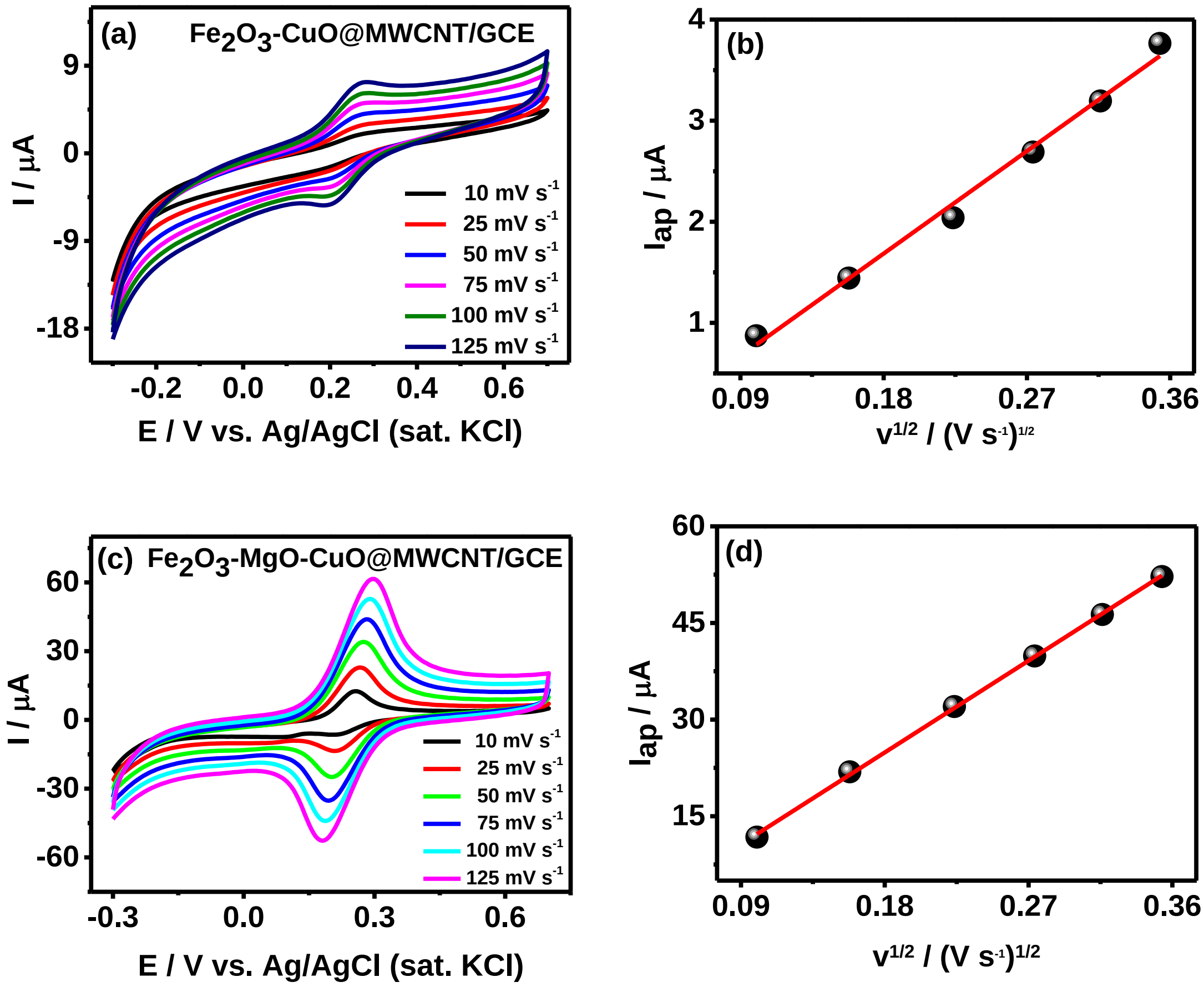


**Figure S2.** Cyclic voltammograms obtained at different scan rates and corresponding plots of anodic peak current (Ipa) versus the square root of scan rate ($\nu^{1/2}$) for (a, b) $Fe_2O_3$–CuO@MWCNT/GCE and (c, d) $Fe_2O_3$–MgO–CuO@MWCNT/GCE, respectively. Measurements were performed using 5 mmol $L^{-1}$ $K_3[Fe(CN)_6]$ as the redox probe in 0.1 mol $L^{-1}$ KCl supporting electrolyte at scan rates of 10, 25, 50, 75, 100, and 125 mV $s^{-1}$.

The electroactive surface areas were estimated from plots of the Iap versus $\nu^{1/2}$ using the Randles–Ševčík equation, considering $n = 1$, $D = 7.6 \times 10^{-6}$ $cm^2$ $s^{-1}$, and $C = 5.0 \times 10^{-6}$ mol $cm^{-3}$.
